# Tobacco and vaping exposure among Spanish adolescents: An analysis of digital, social, school, and family environments

**DOI:** 10.18332/tid/209451

**Published:** 2025-10-31

**Authors:** Cristina Sota Rodrigo, María-Camino Escolar-Llamazares, Elvira Isabel Mercado Val, María Consuelo Sáiz-Manzanares, María Ángeles Martínez Martín

**Affiliations:** 1 Department of Health Sciences, University of Burgos, Burgos, Spain; 2 Department of Educational Sciences, University of Burgos, Burgos, Spain

**Keywords:** passive exposure, secondhand smoke, electronic cigarette aerosol, adolescents, social models, tobacco experimentation, vaping

## Abstract

**INTRODUCTION:**

Involuntary exposure to secondhand tobacco smoke (SHS) and secondhand aerosol from electronic cigarettes (SHA) persists in homes, vehicles, educational settings, and recreational spaces, increasing adolescents’ risk of respiratory infections, asthma, and impaired lung development^1^. The study aim was to examine among Spanish adolescents, aged 12–21 years, the associations between: 1) the presence of social models who smoke or vape (parents, siblings, peers, teachers); 2) self-perceived exposure to smoke or aerosol in physical environments (home, school, car, public spaces); 3) digital exposure to both anti-tobacco messaging and vaping-related content on social media and video platforms; and 4) age-based sales restrictions for nicotine products. We hypothesized that higher levels of physical or digital exposure and the presence of smoking or vaping role models would be associated with greater likelihood of trying conventional or electronic cigarettes.

**METHODS:**

We conducted a cross-sectional survey of 2823 students (mean age=13.8 ± 1.2 years; 49.2% female) in public and charter schools between 2021 and 2024. A validated questionnaire (Cronbach’s α=0.72–0.84) assessed experimental tobacco and vaping use, social models, physical and digital exposures, and purchase attempts/denials. Analyses included bivariate tests (χ^2^, Cramér’s V), logistic regression for tobacco experimentation and multiple linear regression for vaping.

**RESULTS:**

Among participants, 21% had tried cigarettes and 8.3% had used e-cigarettes. Tobacco experimentation was significantly associated with having smoking friends (adjusted odds ratio, AOR=4.47; 95% CI: 3.30–6.06), smoking siblings (AOR=1.87; 95% CI: 1.32–2.64), and exposure to smoking at school (AOR=1.87; 95% CI: 1.39–2.50) or concerts (AOR=1.83; 95% CI: 1.21–2.77). Conversely, exposure at beaches or swimming pools was linked to lower odds (AOR=0.54; 95% CI: 0.36–0.82). E-cigarette use was positively associated with exposure to anti-tobacco media messages (β=0.264, p<0.001), vaping content in online videos (β=0.098, p=0.021), and having smoking friends (β=0.118, p=0.038). Each β indicates the estimated increase in the normalized vaping score per unit increase in the corresponding exposure. Additionally, being denied nicotine product purchases due to age restrictions was linked to greater odds of e-cigarette experimentation (AOR=2.87; 95% CI: 1.94–4.23).

**CONCLUSIONS:**

Τhe findings suggest that family and peer models, as well as passive exposure in both physical and digital environments, may be associated with adolescent initiation of tobacco and vaping. These associations highlight the importance of conducting further longitudinal studies to explore causal mechanisms and inform the development of effective prevention strategies tailored to adolescents’ social and digital contexts.

## INTRODUCTION

Secondhand smoke (SHS) remains one of the most significant threats to public health globally. In 2004, SHS caused more than 600000 deaths and approximately 10 million disability-adjusted life years, with no safe level of exposure for non-smokers^1^. It is classified as a human carcinogen and contains numerous carcinogenic, teratogenic, irritant, toxic, and mutagenic compounds; its inhalation has been linked to cancer, cardiovascular and respiratory diseases in non-smoking adults, as well as otitis media and other respiratory disorders in children^2-4^. According to the most recent WHO report on the global tobacco epidemic, SHS remains responsible for over 1.3 million deaths annually, and although roughly 75% of the world’s population is covered by at least one ‘best-practice’ policy of the MPOWER package, the six evidence-based measures promoted by WHO to support implementation of the Framework Convention on Tobacco Control, substantial variation persists across countries, particularly in warning about its dangers and in universal protection; many have yet to adopt the full set of recommended measures, and some continue to allow tobacco sales without effective restrictions^1^. Available evidence suggests that tobacco control restrictions not only protect health by reducing exposure to pollutants but also re-enforce social norms around tobacco use^5^.

During adolescence, a critical period of physical, emotional, and social development, exposure to SHS poses an added challenge: it is estimated that about 63% of adolescents worldwide have been exposed, contributing to approximately 603000 annual deaths, 28% of which occur among those under 15 years of age^6^. Adolescents are particularly vulnerable to secondhand smoke for biological reasons (higher respiratory rate and immature systems) and psychosocial reasons (less control over their environment), and exposure is unevenly distributed, being more frequent in lower socioeconomic contexts where parental smoking prevalence is also higher^7^ . In addition, SHS during this stage has been associated with increased risk of depression, sleep disturbances, suicide attempts, respiratory infections, obesity, and with the normalization of smoking that facilitates initiation; early initiation of tobacco use appears to increase the likelihood of dependence and premature mortality^8^, being highly exposed in everyday settings such as the home, car, or school entry area^9^.

In Madrid, outdoor hospitality terraces maintain nicotine concentrations (median=0.42 μg/m^3^, IQR: 0.14–1.59) and PM2.5 (median=10.40 μg/m^3^, IQR: 6.76–15.47), significantly above urban background levels, especially when areas are fully enclosed or when tobacco odor and multiple lit cigarettes are simultaneously present^10^. Living with adult smokers or being exposed in the school environment reinforces the idea that smoking or vaping is a common or harmless behavior, reducing risk perception and increasing behavioral vulnerability^11^.

Along the same lines, schools are strategic settings for prevention, not only because of the time young people spend there but also because of their capacity to establish norms and social interactions. School smoke-free policies reduce direct exposure and increase adolescents’ social support for broader restrictions on use^12^. However, certain border areas, such as outdoor school entrances, have typically fallen outside clearly defined regulatory or signage perimeters, even though exposure there, while brief, is repeated daily and accumulates, and observing smoking by reference models plays an important modeling role, functioning as signals of social normalization of use^13^.

Social environments operate as learning spaces: cohabitation with or visible exposure to adults and peers who smoke or vape reinforces the perception that those behaviors are normalized or harmless, facilitating their adoption through social learning^14^.

Concurrently, the emergence of electronic nicotine delivery devices, also known as electronic cigarettes or, colloquially and imprecisely, as ‘vapers’, has raised public health concerns due to their impact on adolescent populations^15^. The secondhand aerosol (SHA) generated by these electronic cigarettes contains toxic and potentially carcinogenic compounds such as acrolein, formaldehyde, and glycidol; it has been detected in indoor environments and shown to expose non-users to elevated biomarkers of exposure^16^ supporting its inclusion in smoke-free air regulations.

Outdoor school entrances are particularly relevant in this context: although exposure there is transitory, its daily repetition over school years creates an accumulated burden, and the visibility of use acts as a normalization cue that can influence future attitudes and behaviors^17^. A multicenter cross-sectional study in 11 European countries documented that those entrances, often excluded from smoke-free policies, harbor substantial SHS exposure and other indicators of use: nicotine was detected in 45.9% of access points, people smoking in 43.2%, and cigarette butts in 75%; exposure was more intense in contexts with lower tobacco control, higher national smoking prevalence, and in areas of lower socioeconomic status, suggesting structural inequities^18^.

In Spain, the ESTUDES surveys indicate that adolescents remain exposed to smoke in multiple settings and that such exposure is associated with greater experimentation and regular use^19^. However, studies simultaneously integrating the physical (specific places), social (models who smoke or vape), and regulatory dimensions of exposure remain scarce.

### Objective and hypothesis

This cross-sectional study analyzes, in a representative sample of secondary school students in the province of Burgos, the associations between the presence of social models who smoke or vape (such as parents, teachers, or peers), passive exposure to tobacco and e-cigarette use in both physical and digital environments, and the perceived ease of access to nicotine products despite legal age restrictions. The sample is composed mainly of adolescents aged 12–21 years. The study hypothesizes that each of these dimensions, environmental exposure, social modeling, and perceived access, is positively associated with experimentation with both conventional and electronic cigarettes.

## METHODS

### Study design

We conducted a cross-sectional, descriptive, and correlational study within the framework of a health education and tobacco prevention program implemented in secondary schools in the province of Burgos, Spain, between 2021 and 2024.

The study examined the associations between the presence of smoking and vaping role models (e.g. parents, siblings, peers, or teachers) and adolescents’ self-perceived exposure to environments with tobacco smoke or e-cigarette aerosol, both in physical and digital settings.

### Sample

The study was conducted with students enrolled in secondary education, vocational training, and Bachillerato (upper secondary/pre-university) in the province of Burgos, Spain. Participants took part in a tobacco prevention and health promotion program between 2021 and 2023. Schools voluntarily adhered to the program and agreed to collaborate in its post-implementation evaluation, resulting in a non-probabilistic convenience sample.

The analytic sample consisted of students aged 11–21 years (mean=13.7; SD=1.5), with the vast majority (>95%) aged between 12 and 17 years, consistent with the developmental stage of adolescence. Participants aged >21 years were excluded from analysis, in line with neurodevelopmental definitions of adolescence.

In terms of educational level, 46.9% of students were in 1st year of compulsory secondary education (ESO), 23.2% in 4th year of ESO, 4.6% in 3rd year of ESO, 0.9% in 2nd year of ESO, and smaller proportions were enrolled in basic vocational training (1.2%), medium and higher level vocational cycles (0.8% and 1.1%, respectively), and Bachillerato (0.5% in 1st year and 0.1% in 2nd year).Gender distribution was 49.2% female, 46.4% male, and 4.4% non-binary. Regarding school type, 60.2% of students attended publicly funded private (concertado) schools and 39.8% attended public schools.

Participation was voluntary and anonymous. Informed consent from parents/legal guardians and assent from adolescents were obtained following procedures managed by the participating schools. The evaluation was conducted within the framework of institutional educational improvement and was thus exempt from formal ethics committee review. Data collection procedures ensured strict confidentiality.

### Instrument

An *ad hoc* questionnaire, specifically developed for this study, was designed based on the TQS-Youth module of the Global Tobacco Surveillance System, a standardized tool developed by the WHO and CDC to assess tobacco-related behaviors in adolescents, as well as the instrument developed by Smart and colleagues (1980).

The covariates included in the analysis were selected based on prior research on adolescent tobacco and e-cigarette use. These variables encompassed: age (years), gender (female, male, non-binary), school type (public vs publicly funded private), and residence (urban vs rural). Additional psychosocial and contextual variables were also considered: perceived social pressure to smoke or vape (yes, no), presence of family or peers who smoke or vape (yes, no), exposure to anti-tobacco messages in the media (Likert-type scale), and participation in school-based prevention programs (yes, no). Finally, we included digital exposure variables, such as frequency of encountering tobacco- or vaping-related content on social media, and perceived social acceptability of vaping and smoking, assessed using composite indicators.

The *ad hoc* questionnaire also included dichotomous items assessing lifetime use of other substances, specifically alcohol and cannabis (0=no, 1=yes), along with age of initiation and context of use. These variables were included to explore potential comorbid patterns and associated contextual factors. Additional items explored the frequency of use and social context (e.g. with friends, at home, during leisure), allowing a broader characterization of adolescent substance use behaviors.

The final version of the instrument was validated through expert judgment and a pilot test (n=154), yielding Cronbach’s alpha values ranging from 0.72 to 0.84 across the different sections.

### Procedure

Data collection was conducted after the implementation of a school-based prevention program, which took place during regular class hours between 2021 and 2024 in secondary schools in the province of Burgos (Spain). This program, delivered by healthcare professionals, consisted of three sequential one-hour modules using active methodologies such as oral presentations, audiovisual materials, group dynamics, and student-led presentations. Module 1 addressed health risks associated with tobacco use and nicotine delivery devices, fostering critical reflection on early initiation. Module 2 focused on the development of assertiveness skills and strategies to cope with peer pressure. Module 3 encouraged critical analysis of advertising and marketing strategies used by the tobacco industry, particularly regarding emerging products such as electronic cigarettes, pods, and vapes.

Once the sessions were completed, families were informed about the evaluation process, as previously announced at the beginning of the academic year. Student participation was voluntary, anonymous, and based on informed consent, obtained in accordance with each institution’s procedures (printed or digital format). Teachers, previously trained for this purpose, administered the questionnaire in the classroom during tutorial periods without intervening in the responses. The questionnaire was applied in paper format during the 2021–2022 academic year and in online format (Google Forms) during the 2022–2023 and 2023–2024 academic years. The average completion time was approximately 13 minutes.

The instrument used was an *ad hoc*, self-administered questionnaire entitled ‘Attitudes and Behavior toward Tobacco among Secondary Education Students’. It consisted of 42 items: 40 categorical (with 2 to 6 response options) and 2 final items using Likert-type scales. The variables were grouped into four main categories: sociodemographic – age (years), gender (boy, girl, other), type of school (public, private), academic year, and residence (urban, rural); substance use – age of onset and frequency of use of tobacco, vapes, alcohol, and cannabis, number of cigarettes smoked weekly, and use of other forms (roll-your-own, hookahs, joints); and risk factors – passive exposure in various settings (home, school, public spaces, car), peer pressure (dichotomous: yes, no), presence of smoking role models (parents, teachers, friends), exposure to social media and vaping-related content (TikTok, Instagram, YouTube), perceived accessibility of tobacco (e.g. ability to purchase without age verification), and perceived self-efficacy to resist social pressure.

The main dependent variables were experimentation with conventional cigarettes (yes, no) and experimentation with electronic cigarettes or vapes (yes, no).

Both were treated as dichotomous variables. Most explanatory variables were categorical and dichotomous, except for those measured on continuous scales (e.g. age, number of cigarettes, self-efficacy or belief scores).

The questionnaire design was based on the World Health Organization’s recommendations for youth tobacco surveys (Smart and colleagues 1980), as well as on prior instruments used by the research team. Content validity was ensured through review by five experts in the health and educational fields. Internal consistency was assessed in previous studies, with Cronbach’s alpha values of α=0.620 (2011), α=0.604 (2016), and α =0.721 (2019), considered acceptable for exploratory research in school populations.

### Data analysis

All statistical analyses were conducted using SPSS version 26 and Jamovi version 2.4. First, we described the characteristics of the sample, the presence of smoking role models, patterns of tobacco use, and passive exposure to tobacco smoke or e-cigarette aerosol. Categorical variables (e.g. gender, school type, exposure settings) were summarized using frequencies and percentages, while the continuous variable (age) was described using mean and standard deviation.

We explored the bivariate association between cigarette experimentation (yes, no) and each form of passive exposure in the following settings: home, school, car, terraces, restaurants, queues, concerts, outdoor spaces, beaches, pools, sports venues, and absence of exposure. Pearson’s chi-squared test (χ^2^) was used, and effect sizes were calculated using Cramér’s V or phi (for 2×2 tables), interpreted according to Cohen’s (1988) guidelines: small (about 0.10), medium (about 0.20), or large (≥0.30) effects.

To identify factors independently associated with cigarette experimentation, we conducted a binary logistic regression analysis using the Enter method, with cigarette experimentation as the dependent variable (0=no, 1=yes). Included in the model was the presence of smoking role models as a factor independently associated with the outcome (father, mother, siblings, uncles/aunts, cousins, teachers, friends, classmates, none), passive exposure in various settings, perception of anti-smoking messages in the media, age-related sales restrictions, use of social media, and exposure to vaping content on media and networks. This logistic model was used exclusively for dichotomous outcomes (e.g. cigarette experimentation). In contrast, multiple linear regression models were applied to continuous dependent variables, such as the overall passive exposure index, as described in later sections.

The model controlled for age, gender, and school type. Model fit was assessed using the Omnibus Test of Model Coefficients and the Hosmer-Lemeshow goodness-of-fit test. We reported Cox & Snell and Nagelkerke pseudo-R^2^ values and the percentage of correctly classified participants. Only odds ratios (ORs) with p<0.05 and their 95% confidence intervals were interpreted.

To compare the discriminative capacity of each passive exposure indicator in distinguishing between experimenters and non-experimenters, we calculated the area under the receiver operating characteristic (ROC) curve (AUC) for each categorical variable. Pairwise comparisons were performed against the ‘smoking at home’ variable using DeLong’s test for paired samples. For all statistical tests, the significance level was set at p<0.05 (two-tailed).

Finally, prior to conducting multivariate analyses, we examined the pattern and extent of missing data. Missing values for key variables are reported in Supplementary file Table 1, all below 1.2%. Little’s MCAR test indicated that data were missing completely at random (p>0.05), so a complete-case analysis was deemed appropriate. The final analytic sample included participants with complete information on all relevant variables. The full sample selection process is detailed in Supplementary file Figure 1.

## RESULTS

Two main dependent variables were defined: 1) experimentation with conventional cigarettes (yes, no) and 2) experimentation with e-cigarettes or vapes (yes, no). Exposure variables included: 1) passive exposure to tobacco smoke or aerosol in various settings (home, school, car, public venues); 2) the presence of smoking or vaping role models (family members, teachers, peers); and 3) exposure to preventive or promotional content, such as reception of anti-smoking messages in the media, age-related purchase restrictions, use of social media, and exposure to vaping content online. Covariates were age, gender, type of school (public vs private), and area of residence (urban vs rural), in line with previous literature on adolescent substance use.

Among the 2240 questionnaires collected, 20.7% contained at least one missing value in key variables. As the pattern of missingness was assumed to be Missing at Random (MAR), we conducted a complete records analysis, retaining only individuals with full data on the relevant outcome and exposure variables

### Role models, substance use, and passive exposure

A large proportion of adolescents (83.2%) reported regular exposure to at least one smoker in their immediate environment. The most frequently cited sources were extended family members (49.4%), followed by friends (33.8%), classmates (33.4%), and teachers (32.8%). At home, 21.3% reported that their father smoked, 17.8% their mother, and 6.6% a sibling. Only 16.5% reported no exposure in any setting.

Regarding substance use, 21.0% had experimented with cigarettes, with a mean initiation age of 13.66 years. Vaping experimentation reached 8.3%. In the past month, 10.3% reported vaping, 7.6% had smoked cigarettes, 5.4% used roll-your-own tobacco, 3.9% consumed cannabis joints, and 3.3% used hookahs. Daily smoking was reported by 4.5%, and 1.8% consumed more than 20 cigarettes per day. When asked about future expectations, 76% did not believe they would smoke in five years, 4.8% believed they would, and 19.2% were uncertain.

As for perceived social pressure, 57.1% reported having friends who vaped, and 13.2% had experienced pressure to smoke or vape. Despite this, 59.0% felt highly capable of resisting peer influence, while only 4.0% reported low resistance.

**Figure 1:**
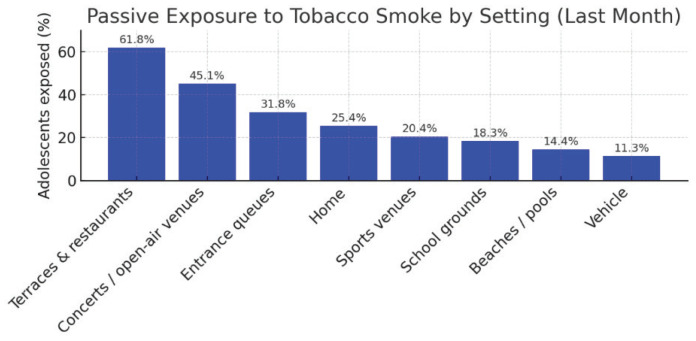
Percentage of adolescents passively exposed to tobacco smoke in different settings during the past month, a cross-sectional study conducted among secondary school students in Spain, 2021–2023 (N=2240)

Passive exposure to tobacco smoke remained widespread. In the past month, 61.8% had been exposed on terraces or in other outdoor leisure venues, 45.1% at public events (e.g. concerts), and 31.8% while queuing. Exposure at home was reported by 25.4%, followed by school (18.3%) and private vehicles (11.3%). Only 16.5% reported no exposure in any setting, confirming the persistence of involuntary exposure in both public and private environments (Figure 1).

### School exposure, access, and preventive messages

**Figure 2:**
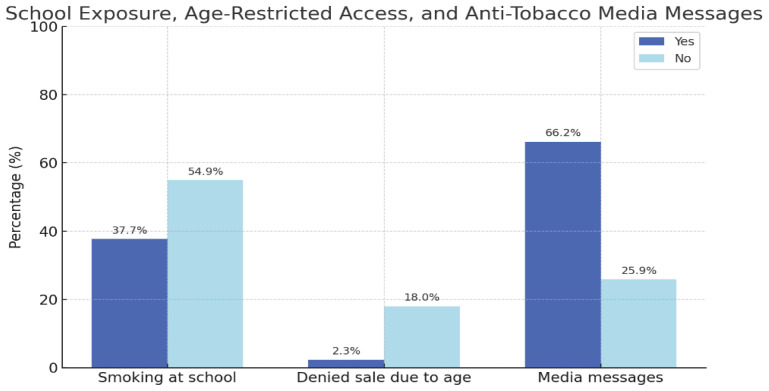
Prevalence of school-based exposure to smoking, age-restricted sales enforcement, and recall of anti-tobacco media messages among adolescents, a cross-sectional study conducted among secondary school students in Spain, 2021–2023 (N=2240)

A total of 37.7% of participants reported having witnessed someone smoking inside or on school premises during the past month, suggesting limitations in the enforcement of smoke-free policies in educational settings (Figure 2). While 54.9% stated they had not observed such behavior, 7.4% selected a less specific option (‘more than once’), which may warrant refinement in future survey designs to improve clarity regarding the context, frequency, or location of smoking behavior. Regarding access, 18.0% of adolescents indicated that age restrictions did not pose a barrier when purchasing cigarettes, whereas only 2.3% reported having been explicitly denied a sale. The majority (79.8%) did not attempt to purchase tobacco or vaping products during the previous month, which may reflect access through peers or low motivation to initiate purchase.

In terms of prevention, 66.2% of students reported being exposed daily to anti-tobacco messages, and 7.3% at least once per week. However, 25.9% indicated not having encountered any such messages in the past month, revealing gaps in the consistency and coverage of media-based prevention efforts. Although frequent exposure to preventive messages may contribute to health-promoting attitudes, its impact likely depends on additional contextual and social factors.

### Bivariate and multivariate analysis of tobacco experimentation

Pearson’s χ^2^ tests were used to examine the association between tobacco experimentation (ever tried: yes, no) and passive exposure to smoking across various settings during the past month. As shown in Table 1, most environments showed statistically significant associations with having tried cigarettes, with small to moderate effect sizes. The strongest associations were observed for exposure at school (Cramér’s V=0.211), at home (V=0.188), and in private vehicles (V=0.190). In contrast, no significant association was found for beaches or swimming pools (p=0.121). Importantly, adolescents who reported no exposure in any setting were significantly less likely to have tried tobacco (inverse association: V= -0.135).

**Table 1:** Association between exposure to smoking environments and having ever tried cigarettes, a cross-sectional study conducted among secondary school students in Spain, 2021–2023 (N=2240)

*Setting*	*χ² (1)*	*p*	*Cramér’s V*	*Effect size*
Home	79.39	<0.001	0.188	Small–moderate
School	99.27	<0.001	0.211	Moderate
Car	80.50	<0.001	0.190	Small–moderate
Terraces/restaurants	14.65	<0.001	0.081	Small
Entrance queues	15.58	<0.001	0.083	Small
Concerts/open-air venues	67.35	<0.001	0.173	Small–moderate
Beaches/pools	2.40	0.121	0.033	Not significant
Outdoor sports venues	29.01	<0.001	0.114	Small
None (inverse exposure)	40.95	<0.001	-0.135	Small (inverse)

Effect sizes were interpreted using commonly accepted thresholds for Cramér’s V: small (0.06–0.17), moderate (0.18–0.29), strong (≥0.30). Negative values indicate inverse associations.

To explore the contribution of a broader set of contextual and psychosocial variables, a multiple linear regression analysis was conducted. The outcome variable was a dichotomous measure of tobacco experimentation (0=never tried, 1=ever tried). The model included 29 variables related to social environment, access, beliefs, media exposure, and self-efficacy. Despite the binary outcome, the linear regression was used to estimate standardized coefficients and explore relative contributions under robust assumptions, following previous approaches in similar cross-sectional designs. The model explained 29.4% of the variance in tobacco experimentation (adjusted R^2^=0.283), with a satisfactory fit (R=0.542; F(29, 1788)=25.699; p<0.001). Full regression coefficients and diagnostics are available upon request.

### Multivariate analysis of tobacco experimentation

Most of the environments analyzed showed a significant association with having ever tried tobacco (p<0.001). The strongest effect size was observed for exposure at school (Cramér’s V=0.211), followed by exposure at home (V=0.188) and in private vehicles (V=0.190). No significant association was found with exposure at beaches or swimming pools (p=0.121). Interestingly, adolescents not exposed to smoking in any setting were significantly less likely to have tried tobacco, suggesting a protective association (V= -0.135). According to Cohen’s guidelines (1988), effect sizes ranged from small (φ about 0.10) to moderate (V=0.211).

To explore the factors most strongly associated with tobacco experimentation, we conducted a multiple linear regression using the Enter method and including 29 variables related to family, peers, exposure settings, and media influences. Although the outcome variable was dichotomous (ever tried tobacco: yes, no), linear regression was used to estimate standardized coefficients and compare the relative strength of associations, as has been done in prior cross-sectional studies. The model was statistically significant [F(29, 1788)=25.699, p<0.001], with an acceptable fit (R=0.542; R^2^=0.294; adjusted R^2^= 0.283), explaining nearly 29% of the variance in tobacco experimentation.

Significant associations were found with sibling smoking (p<0.001), peer smoking (p<0.001), and the absence of any smoker at home (p=0.031); exposure to smoking at school (p<0.001), in cars (p=0.034), and at concerts or open-air events (p<0.001), with a negative association for exposure at beaches or swimming pools (p=0.002); as well as exposure to vaping content in music videos (p=0.001), on Instagram (p=0.001), and frequent media use (p=0.001). Exposure to anti-tobacco messages did not show a significant association.

To complement these findings and obtain a more precise estimate of the probability of tobacco experimentation, we also conducted a binary logistic regression with the same set of variables. This approach, more appropriate for a dichotomous outcome, allowed the calculation of odds ratios (ORs) and confidence intervals. The results of this logistic regression model are presented in Table 2.

**Table 2:** Multiple linear regression coefficients predicting the likelihood of having tried cigarettes by setting of exposure, a cross-sectional study conducted among secondary school students in Spain, 2021–2023 (N=2240)

*Variable*	*B*	*SE B*	*β*	*t*	*p*	*95% CI for B*
Constant	-0.135	0.040		-3.343	0.001	(-0.214 – -0.056)
**Family smoking environments**						
Father	0.048	0.024	0.047	2.024	0.043	(0.001–0.09)
Mother	0.040	0.026	0.036	1.553	0.121	(-0.010–0.090)
Sibling	0.140	0.036	0.083	3.907	<0.001	(0.070–0.210)
Uncles/cousins	0.016	0.019	0.019	0.823	0.411	(-0.022–0.053)
Teachers	-0.006	0.019	-0.007	-0.334	0.738	(-0.044–0.031)
Friends	0.238	0.021	0.270	11.581	<0.001	(0.198–0.279)
Classmates	0.028	0.020	0.031	1.377	0.169	(-0.012–0.067)
No smoker present	0.061	0.028	0.054	2.164	0.031	(0.006–0.115)
**Exposure settings** (past month)						
Home	0.027	0.025	0.029	1.077	0.282	(-0.022–0.077)
School	0.112	0.024	0.103	4.631	<0.001	(0.064–0.159)
Car	0.065	0.031	0.050	2.116	0.034	(0.005–0.126)
Terraces/restaurants	-0.013	0.022	-0.015	-0.592	0.554	(-0.057–0.030)
Entrance queues	-0.037	0.021	-0.042	-1.792	0.073	(-0.078–0.004)
Concerts/open-air	0.083	0.021	0.098	3.875	<0.001	(0.041–0.125)
Beaches/pools	-0.084	0.027	-0.070	-3.155	0.002	(-0.136 – -0.032)
Outdoor sports venues	-0.020	0.024	-0.020	-0.840	0.401	(-0.068–0.027)
No setting	-0.048	0.029	-0.043	-1.635	0.102	(-0.105–0.009)
School grounds	-0.004	0.013	-0.007	-0.318	0.751	(-0.031–0.022)
Age restriction enforced	0.172	0.020	0.193	8.797	<0.001	(0.134–0.211)
Anti-tobacco messages	-0.002	0.017	-0.002	-0.100	0.921	(-0.034–0.031)
**Media exposure to vaping**						
Mass media	-0.029	0.023	-0.030	-1.273	0.203	(-0.074–0.016)
Music videos	0.080	0.024	0.081	3.287	0.001	(0.032–0.127)
TikTok	-0.016	0.024	-0.019	-0.666	0.505	(-0.064–0.031)
Instagram	0.078	0.024	0.090	3.310	0.001	(0.032–0.125)
Snapchat	-0.001	0.044	-0.001	-0.025	0.980	(-0.087–0.084)
Other platforms	-0.033	0.031	-0.023	-1.064	0.287	(-0.094–0.028)
Don’t know	0.012	0.028	0.010	0.437	0.662	(-0.042–0.066)
**Social media user**						
Mass media	-0.023	0.007	-0.075	-3.459	0.001	(-0.035 – -0.010)

B: unstandardized coefficient. β: standardized coefficient. SE B: standard error in Β. Method: Enter. Although the dependent variable is dichotomous, standardized coefficients (β) are included for comparative interpretation.

* Significant associations p<0.05.

Exposure to vaping-related videos was positively associated with higher normalization scores (β=0.31, p<0.01), indicating that each additional unit on the exposure scale predicted a 0.31-point increase in the normalization index.

To examine factors associated with having ever tried or attempted to smoke cigarettes (1 = ‘yes’; 0 = ‘no’), a binary logistic regression was performed using the Enter method, incorporating personal, family, and exposure-related variables as factors associated with the outcome. The model demonstrated acceptable fit and explanatory power. The Omnibus Test of Model Coefficients was significant [χ^2^(30)=554.39, p<0.001], and the –2 log-likelihood was 1403.66. Pseudo-R^2^ values were 0.263 (Cox & Snell) and 0.399 (Nagelkerke), indicating a moderate proportion of explained variance. Model calibration was satisfactory, as indicated by the Hosmer-Lemeshow test [χ^2^(8)=9.33, p=0.315].

Overall, the model correctly classified 83.3% of participants, with high specificity (94.3% for non-smokers) and moderate sensitivity (46.3% for those who had tried smoking). These results underscore both the model’s utility and the likely contribution of additional unmeasured psychosocial or contextual factors in predicting tobacco experimentation.

### Factors independently associated with cigarette experimentation

The regression model confirmed the strong influence of peer context and perceived risk on cigarette initiation. Having friends who smoke markedly increased the likelihood of trying cigarettes (AOR=4.47), followed by having a smoking sibling (AOR=2.11) and experiencing sales denial due to age (OR=2.87). Importantly, the belief that ‘vaping is not harmful’ showed the strongest association (AOR=22.88), suggesting a spillover effect from vaping-related risk minimization. In contrast, passive exposure in recreational settings (e.g. beaches or pools) was linked to reduced odds of experimentation (AOR=0.54) (Table 3).

**Table 3:** Adjusted odds ratio (AOR) from binary logistic regression predicting adolescent cigarette experimentation a cross-sectional study conducted among secondary school students in Spain, 2021–2023 (N=2240)

Variable	B	SE	Wald	p	AOR (95% CI)
Sibling smokes	0.746	0.248	9.07	0.003	2.11 (1.30 – -3.43)
Friends who smoke	1.497	0.155	93.55	<0.001	4.47 (3.30–6.06)
Exposure at school (last month)	0.624	0.174	12.82	<0.001	1.87 (1.33–2.63)
Exposure in car (last month)	0.404	0.219	3.40	0.065	1.50 (0.98–2.30)
Exposure at concerts/open spaces	0.605	0.166	13.31	<0.001	1.83 (1.32–2.53)
Refusal to sell due to age (last month)	1.055	0.138	58.37	<0.001	2.87 (2.19–3.77)
Social media use	-0.306	0.072	17.91	<0.001	0.74 (0.64–0.85)
Vaping in music videos (last week)	0.510	0.181	7.92	0.005	1.67 (1.17–2.37)
Vaping on Instagram (last week)	0.591	0.188	9.83	0.002	1.81 (1.25–2.61)
Belief ‘vaping is not harmful’	3.130	1.442	4.72	0.030	22.88 (1.36–385.99)
Beaches/pools (last month)	-0.616	0.212	8.42	0.004	0.54 (0.36–0.82)

AOR: adjusted odds ratio. Dependent variable: having tried conventional cigarettes during adolescence (yes=1, no=0). B: unstandardized logistic regression coefficient. SE: standard error. Wald: Wald chi-squared statistic. Only variables with statistically significant associations (p<0.05) are shown; non-significant variables were omitted (e.g. parental smoking, exposure in restaurants, in queues, or anti-tobacco messages) to ensure model parsimony.

### Factors independently associated with vaping experimentation

To examine the factors associated with experimentation with e-cigarettes, two complementary regression models were conducted. First, a multiple linear regression was applied using a continuous index of vaping experimentation, rather than a binary (yes, no) variable. This index incorporated multiple items on frequency, contexts, and modalities of e-cigarette use. The model was statistically significant [F(29, 1789)=11.17, p<0.001], explaining 15% of the variance (adjusted R^2^=0.140).

Peer smoking (β=0.118) and recent exposure to vaping in open-air settings (β=0.098) were positively associated with higher experimentation levels. Surprisingly, exposure to anti-tobacco messages in the media also showed a strong positive association (β=0.264), possibly reflecting reverse causality or psychological reactance.

In contrast, frequent social media use (β= -0.072), exposure in recreational areas such as beaches or pools (β= -0.071), and viewing vaping content on non-mainstream platforms (β= -0.089) were negatively associated with vaping experimentation, potentially indicating awareness, avoidance, or saturation effects.

All variables met collinearity criteria (variance inflation factor, VIF <2), supporting the model’s stability. The VIF is a statistical indicator used to assess the degree of multicollinearity among independent variables; values >5, especially those >10, are often considered indicative of potential multicollinearity problems.

To complement this analysis and address potential binary interpretations, a logistic regression model was also performed using a dichotomous dependent variable (having tried vaping: yes, no). The results are presented in Table 4.

**Table 4:** Binary logistic regression analysis of vaping experimentation (yes, no), a cross-sectional study conducted among secondary school students in Spain, 2021–2023 (N=2240)

Variable	B	SE B	Wald	df	p	OR (95% CI)
Friends who smoke	1.50	0.156	92.33	1	<0.001	4.47 (3.30–6.06)
Siblings who smoke	0.75	0.255	8.63	1	0.003	2.11 (1.30–3.43)
Refused tobacco sale (underage)	1.05	0.140	56.64	1	<0.001	2.87 (2.19–3.77)
Seen smoking at school	0.63	0.120	27.60	1	<0.001	1.87 (1.48–2.37)
Exposure at concerts	0.60	0.130	21.22	1	<0.001	1.83 (1.42–2.36)
Exposure on beaches/pools	-0.62	0.217	8.52	1	0.004	0.54 (0.36–0.82)
Belief: vaping not harmful	3.13	1.45	4.67	1	0.030	22.88 (1.34–390.81)
Exposure: music videos	0.51	0.150	11.53	1	<0.001	1.67 (1.25–2.23)
Exposure: Instagram	0.59	0.143	17.15	1	<0.001	1.81 (1.38–2.39)
Frequent social media use	-0.30	0.080	14.06	1	<0.001	0.74 (0.63–0.88)

B: unstandardized logistic regression coefficient. SE: standard error. df: degrees of freedom. Only variables with statistically significant associations (p<0.05) are shown.

Logistic regression identified several factors significantly associated with the likelihood of having experimented with e-cigarettes during adolescence. The strongest association was observed for having friends who smoke, which increased the odds more than fourfold (OR=4.47; 95% CI: 3.30–6.06; p<0.001). Additional significant predictors included having a sibling who smokes (OR=2.11), being denied tobacco purchases due to age (OR=2.87), and exposure to smoking at school (OR=1.87) or at concerts (OR=1.83).

Conversely, exposure on beaches or at swimming pools was associated with a lower likelihood of vaping experimentation (OR=0.54; 95% CI: 0.36–0.82; p=0.004). Believing that ‘vaping is not harmful’ showed a strong, albeit imprecise, association (OR=22.88; p=0.030), suggesting a potential cognitive spillover effect. Exposure to vaping-related content in music videos (OR=1.67) and on Instagram (OR=1.81) also increased the odds of trying e-cigarettes, whereas frequent social media use appeared protective (OR=0.74; p<0.001).

Multicollinearity diagnostics confirmed the stability of the model (VIF <2.10; tolerance >0.48; condition index=17.9). Additionally, exploratory ROC analyses suggested that exposure in certain contexts, such as beaches or swimming pools may help distinguish adolescents who have tried tobacco or vaping from those who have not. However, the overall predictive power of these models was modest, as indicated by the AUC (area under the curve) values ranging from 0.60 to 0.66. The AUC is a common indicator that shows how well a model can separate the two groups; values closer to 1 indicate better discrimination.

## DISCUSSION

### Social and familial role models

In our group of 2240 adolescents (13.8 ± 1.2 years; 49.2 % female), youth smoking behavior was strongly shaped by their immediate social circle. Living with friends who smoke was strongly associated with increased likelihood of having tried cigarettes, and sharing a home with smoking siblings was also associated with a greater likelihood of having tried cigarettes These findings are consistent with the literature^20-23^ identifying peer groups as the chief driver of smoking uptake during adolescence, which showed that older siblings’ smoking directly predicts tobacco use among younger siblings, whereas Low et al.^24^ described how sibling collusion and conflict, mediated by affiliation with high-risk peers, facilitates experimentation. More recently, Luu et al.^25^ confirmed that this influence extends to e-cigarette use: having friends who vape was significantly associated with a greater likelihood of both occasional and current use, with consistent effects across all subgroups and particularly marked before the age of 18 years.

Conversely, the presence of smokers in the extended family (uncles and cousins) was not significant, reinforcing the idea that, during adolescence, sibling and close-friend relationships constitute the main socializing agents for smoking initiation.

We also observed that age-based sales restrictions were strongly associated with a higher likelihood of cigarette experimentation among adolescents. However, few recent studies have attempted to quantify this association in detail. To address this gap, secondary analyses of tobacco-access surveys (e.g. GYTS) or field studies that directly record purchase attempts and their consequences on youth behavior would be useful.

### Passive exposure in everyday settings

Our findings confirm that visible tobacco smoke or e-cigarette aerosol in common social settings functions as a potent environmental cue, increasing the likelihood of experimentation during adolescence. Specifically, observing smoking on school grounds, in leisure settings such as concerts or festivals, or inside private vehicles was consistently linked to a higher likelihood of trying cigarettes. These findings align with previous studies. Mantey et al.^26^ described greater exposure to secondhand smoke in private spaces among rural adolescents compared to their urban counterparts, while Yang et al.^27^ highlighted the strong influence of observing teachers smoke at school on students’ environmental exposure and vulnerability to smoking initiation.

Long-term follow-up of 3637 adolescents aged 12–16 years in Minnesota showed that living in a smoke-free home significantly reduces the likelihood of adopting any of the identified smoker profiles, whereas co-residing with smoking adults or holding favorable perceptions of the tobacco is associated with a greater likelihood of progressing to heavier consumption patterns^28^. Despite a decline in passive exposure in the United States between 2011 and 2019, 25.3% of high-school students in 2019 still reported inhaling smoke at home and 23.3% in private vehicles, underscoring that private settings remain critical sources of secondhand smoke^29^.

In Spain, although specific adolescent studies are scarce, the TackSHS project, involving Hospital de La Princesa, estimated that 31% of the non-smoking population is exposed daily to smoke at home, in transport, and in hospitality and leisure venues, suggesting that young people constitute a substantial portion of that group^30^.Consistently, Lipperman-Kreda et al.^31^ showed that each day of exposure to tobacco displays at festivals and concerts was associated with increased experimentation. Similarly, exposure to smoking in public retail settings such as kiosks or convenience stores significantly increased the likelihood of adolescent tobacco use.

### Modeling experimentation with e-cigarettes

The linear model revealed that several psychosocial variables meaningfully contributed to the likelihood that an adolescent had tried an e-cigarette. Among these, frequent exposure to anti-tobacco messages in the media emerged as the most influential factor, followed by having friends who smoke conventional cigarettes and witnessing smoking or vaping in social environments such as concerts or outdoor venues. These findings are consistent with previous research by Margolis et al.^32^ which highlighted the interactive role of advertising, social modeling, and curiosity in promoting youth engagement with e-cigarettes.

### Predictive power and relevance of total absence of exposure

Comparisons of predictive power across different passive exposure settings revealed that no single context strongly discriminated between experimenters and non-experimenters. However, adolescents who reported a complete absence of exposure to smoke or aerosol in any setting consistently demonstrated the lowest likelihood of cigarette experimentation. These findings underscore the importance of comprehensive protection across all environments, not just isolated contexts, as a critical strategy to prevent smoking initiation among youth.

This result aligns with prior evidence supporting the implementation of comprehensive smoke- and aerosol-free policies in all settings, including workplaces, recreational venues, homes, schools, and transportation, as recommended^33^. In this context, peer-led interventions that foster mutual support and refusal skills regarding both combustible tobacco and e-cigarettes remain essential^34^. Likewise, strict monitoring and enforcement in schools, private vehicles, and leisure venues, including outdoor terraces, are necessary to reduce the visibility and social acceptability of smoking.

In parallel, communication campaigns should address the harms of both tobacco and e-cigarettes simultaneously, clearly explaining that both expose users and bystanders to nicotine, toxicants, and ultrafine particles while avoiding messages that may inadvertently spark curiosity about the alternative product^35^. These strategies are aligned with World Health Organization guidelines^36^ and are essential for the denormalization of nicotine consumption and for safeguarding adolescent health.

Taken together, these findings highlight the urgent need for multi-sectoral public health efforts to expand 100% smoke- and aerosol-free environments across all relevant settings. Equally important are family-based programs that promote smoke-free norms at home and limit adolescents’ access to nicotine products. In parallel, evidence-based school interventions should enhance refusal skills and critical awareness regarding both smoking and vaping. Finally, integrated communication strategies targeting both products simultaneously are needed to create a comprehensive prevention and intervention framework that effectively protects youth health.

### Limitations

This study has several limitations. First, the cross-sectional design prevents establishing causal relationships between the variables analyzed, as reverse causality cannot be ruled out (e.g. adolescents who already smoke may be more likely to report certain exposures). Second, all data were obtained through self-reported questionnaires, which introduces the possibility of information bias due to misclassification (e.g. underreporting of socially undesirable behaviors or overestimation of preventive exposures). Additionally, although the regression models included several sociodemographic and contextual covariates, residual confounding from unmeasured variables cannot be excluded, such as parental monitoring, academic performance, or psychological traits like sensation-seeking.

Furthermore, the sample, although large and diverse, was drawn from schools in a specific region of Spain, which may limit the external validity of the findings. Generalizability to other populations, especially in countries with different tobacco control policies, cultural norms, or marketing environments, should be approached with caution. Longitudinal studies are needed to confirm the directionality of the observed associations and to identify potential mediating mechanisms over time.

### Implications

These results suggest that beyond modeling effects, passive exposure to smoke or aerosol in everyday settings may contribute to the normalization of tobacco use and, consequently, to earlier experimentation. Additionally, easy access to tobacco products in Spain, despite legal age restrictions, may amplify this effect, especially in the absence of active enforcement and monitoring.

## CONCLUSIONS

The findings demonstrate that the presence of smoking role models particularly friends and siblings together with passive exposure in a variety of settings is strongly associated with cigarette experimentation and, to a less extent, with e-cigarette use among adolescents. Conversely, the complete absence of exposure showed the strongest independent association with never having tried cigarettes. These results highlight the need for multisectoral interventions that integrate regulatory measures, education, and coordinated communication campaigns aimed at denormalizing all forms of nicotine and aerosol exposure and support the urgent strengthening of public policies and comprehensive prevention programs across multiple settings to effectively reduce adolescent nicotine use. Future longitudinal research is needed to confirm causality and evaluate intervention efficacy.

## Supplementary Material



## Data Availability

The data supporting this research are available from the authors on reasonable request.
