# Supplementary material for: Tobacco and vaping exposure among Spanish adolescents: An analysis of digital, social, school, and family environments
Source: Tob Induc Dis. 2025 Oct 31;23:10.18332/tid/209451. doi: 10.18332/tid/209451 (PMC12579109; doi:10.18332/tid/209451)
Supplement: Supplementary file 1 [file TID-23-167-s1.pdf]

Supplementary Table 1. *Missing Values by Variable*

**Variable**	Missing Values (n)	Missing (%)
Tried Cigarettes (Yes/No)	45	0.8
Tried Vaping (Yes/No)	38	0.7
Friends Who Smoke	61	1.1
Siblings Who Smoke	52	0.9
Exposure at School	43	0.8
Exposure to Vaping Content	37	0.7
Perceived Anti-Tobacco Messages	29	0.5
Age-Based Purchase Denial	41	0.7

Supplementary Figure 1. *Flow diagram of the sample selection process. Number of eligible students, respondents, and valid participants regarding tobacco and vaping use.*

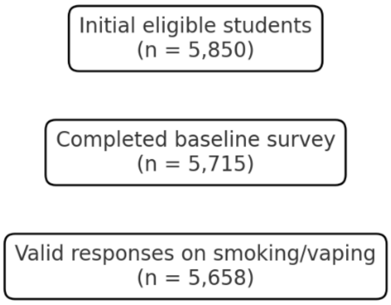

© 2025 Sota Rodrigo C. et al.
